# Rituximab treatment in myasthenia gravis

**DOI:** 10.3389/fneur.2023.1275533

**Published:** 2023-10-02

**Authors:** Ana Vesperinas-Castro, Elena Cortés-Vicente

**Affiliations:** ^1^Neuromuscular Diseases Unit, Department of Neurology, Hospital de la Santa Creu i Sant Pau, Barcelona, Spain; ^2^Department of Medicine, Universitat Autònoma de Barcelona, Barcelona, Spain; ^3^Biomedical Research Institute Sant Pau (IIB Sant Pau), Barcelona, Spain; ^4^Centro de Investigación Biomédica en Red de Enfermedades Raras (CIBERER), Instituto de Salud Carlos III, Madrid, Spain

**Keywords:** myasthenia (myasthenia gravis—MG), Rituximab, refractory patients, efficacy and safety, B-cell depletion

## Abstract

Myasthenia gravis (MG) is a chronic autoimmune disease mediated by antibodies against post-synaptic proteins of the neuromuscular junction. Up to 10%–30% of patients are refractory to conventional treatments. For these patients, rituximab has been used off-label in the recent decades. Rituximab is a monoclonal antibody against the CD20 protein that leads to B cell depletion and to the synthesis of new antibody-secreting plasma cells. Although rituximab was created to treat B-cell lymphoma, its use has widely increased to treat autoimmune diseases. In MG, the benefit of rituximab treatment in MuSK-positive patients seems clear, but a high variability in the results of observational studies and even clinical trials has been reported for AChR-positive patients. Moreover, few evidence has been reported in seronegative MG and juvenile MG and some questions about regimen of administration or monitoring strategies, remains open. In this review, we intend to revise the available literature on this topic and resume the current evidence of effectiveness of Rituximab in MG, with special attention to results on every MG subtype, as well as the administration protocols, monitoring strategies and safety profile of the drug.

## 1. Introduction

Myasthenia Gravis (MG) is a chronic autoimmune disease mediated by antibodies against the acetylcholine receptor (AChR), Muscle-Specific Kinase (MuSK) or other proteins in the neuromuscular junction such as Low-Density Lipoprotein Receptor Related Protein-4 (LRP4). There is a 10%–15% of patients without detectable antibodies in serum, named seronegative MG (1).

The main symptom is weakness, which characteristically get worse with sustained exercise and can affect extraocular, bulbar, limb, and axial muscles. Fifteen percent of patients have only ocular symptoms whereas most of them have a generalized presentation. Respiratory muscles can occur times, with the subsequent need for ventilatory support. This life-threatening situation is called myasthenic crisis and implies a mortality risk of 5%–12% (1–3).

MG therapeutic approach usually includes a combination of symptomatic treatment with acetylcholinesterase inhibitors which increase acetylcholine levels at the neuromuscular junction, thymectomy in selected patients and long-term immunosuppressive medications, with a wide range of options which goes from conventional agents to new immunomodulatory therapies (4, 5). Corticosteroids remain the first-line treatment but long-term use is limited by the burden of adverse events. Immunosuppressants, such as Azathioprine, Mycophenolate Mofetil, Cyclosporine or Tacrolimus are essential for reducing prednisone to the lowest possible dose and prevent relapses.

Biologic therapies have emerged in the last decades with a highly selective target and better security profile than classic immunosuppressants. Rituximab leads to depletion of B cells and its use remains off-label. Two recently developed treatment strategies are complement blockade (such as Eculizumab, Ravulizumab, and Zilucoplan) and neonatal Fc receptor (FcRn) antagonism (Efgartigimod and Rozanolixizumab) (5, 6).

Thanks to all these therapeutic advances, MG prognosis has markedly improved in the last decades. A multicenter study in Norwegian population did not find any increased mortality in patients with MG compared with controls (7).

Despite this, most patients do not reach complete clinical remission, and they do persist symptomatic or they need lifelong immunosuppressing treatments to control the disease. Moreover, there are a 10%–30% of patients with refractory MG, which do not respond to therapies (2, 8).

Rituximab (RTX) has been postulated as a therapeutic option in refractory MG. However, there are some controversies in literature and unresolved questions, such as its efficacy in every serological group, regimen of administration or monitoring to decide retreatment. Recently, several studies including two clinical trials have been published, providing new evidence to this matter.

In this paper, we aim to review the current evidence of the use of Rituximab in MG, with special attention to the serological subtypes.

## 2. Mechanism of action

Rituximab is a human/murine chimeric monoclonal antibody against the CD20 protein, administered via intravenous infusion (9).

It is a molecule composed of the CD20-recognizing regions of murine origin, fused to the constant region of the heavy chain of human IgG1 and human kappa light chain.

CD20 is a glycosylated transmembrane phosphoprotein present on the surface of developing B lymphocyte cells, while progenitor cells and mature plasma cells do not express this marker. Although its cellular function is not fully understood, it is believed to participate in cellular development and activation processes through the regulation of transmembrane calcium flux (10).

Its limited expression in intermediate stages of B cell maturation, but not in progenitor or mature cells or other normal cell lines, makes CD20 an effective and safe potential therapeutic target without permanent side effects.

The binding of the monoclonal antibody to the CD20 receptor induces cell death through four different mechanisms, three of which are dependent on the patient’s immune system: antibody-dependent cellular cytotoxicity through the activation of NK cells, complement-dependent cytotoxicity through cascade activation and, finally, membrane attack complex-dependent and antibody-dependent phagocytosis through macrophage activation. The last mechanism is independent of the immune system and is based on the activation of intracellular mechanisms such as the caspase pathway and lysosomal activation, leading to cell apoptosis (9, 10).

This leads to depletion of circulating B lineage cells and, therefore, the synthesis of new antibody-secreting plasma cells.

Despite the main function of B lymphocytes being the production of antibodies, in recent years, other functions of these cells have been recognized. On one hand, B cells play a role as antigen-presenting cells through the major histocompatibility complex type II to CD4+ Th lymphocytes, participating in their activation alongside dendritic cells. Another important function is cytokine secretion. Regulatory B cells are a subset of B cells that contribute to inflammation control by secreting IL-10, promoting the differentiation of CD4 T lymphocytes into regulatory T cells. Various studies have demonstrated the benefit of B cell depletion therapies in autoimmune diseases mediated by self-reactive T cells (11–13).

## 3. Drug history and indications

Rituximab is the first monoclonal antibody implemented in oncology and remains the most widely used to this day. It was created by Ronald Levy with the goal of targeting malignant B cells, and in 1982, the first case of a successfully treated cancer patient with this antibody was published. In 1994, the first phase I clinical trial of rituximab was conducted in patients with Non-Hodgkin’s lymphoma (10). This led to FDA approval in the United States in 1997 for the treatment of Non-Hodgkin’s lymphoma. Since then, it has been approved for other indications such as rheumatoid arthritis (2006), Wegener’s granulomatosis, microscopic polyangiitis (2011), chronic lymphocytic leukemia (2017), and pemphigus vulgaris (2018) (14).

However, Rituximab is used off-label for numerous indications. In a retrospective study conducted in the United States that reviewed Rituximab administration indications, an increase in off-label indications was observed from 1.2% in 2009 to 55.6% in 2017 (15).

In this same study, the main off-label indication was neurological diseases, including multiple sclerosis, other inflammatory CNS diseases, neuropathies such as CIDP, Stiff-Person syndrome, refractory MG, among others (15, 16).

## 4. Use of rituximab in myasthenia gravis

The first reported case of Rituximab use in MG was in 1999, in a 27-year-old patient who developed refractory myasthenia gravis after a hematopoietic stem cell transplant in the context of acute non-lymphocytic leukemia. The patient experienced improvement in myasthenia gravis, supporting the efficacy of Rituximab in other autoimmune disorders mediated by autoantibodies (17). Since then, the use of rituximab in MG has been widely extended to patients with refractory disease (2, 18) and various articles have reported its efficacy in up to 50%–84%, depending on the report (5, 19–21).

Several series of cases were reported at the beginning of the 2000s. In 2008, Isabel Illa et al. published an observational study including their experience with 6 patients with refractory MG (22). In the next years, several retrospective and prospective studies came out, including a wider number of patients (20, 23–25). In 2014 was published the first systematic review (19) and other meta-analysis have appeared later (26, 27). Recently, in 2022 two clinical trials have been published to increase evidence about this subject.

Table 1 resumes the main characteristics of previous studies.

**Table 1:** Resume of characteristics of previous studies.

References	Sample size (AChR/Musk/SNMG)	Follow-up after RTX, mean, months	RTX regimen	Primary outcome	Result
**Clinical trials**					
Nowa et al. (28)	52 (52/0/0)	13	2 cycles of 375 mg/m^2^ × 4 weeks every 6 months.	Steroid-sparing effect.	Futility (60% with RTX vs. 56% with placebo).
Piehl et al. (29)	47 (45/0/2)	12	Single infusion of 500 mg.	Minimal disease manifestations (QMG < 4 or prednisolone < 10 mg/day).	Positive (71% with RTX vs. 29% in placebo arm).
**Meta-analysis**					
Feng et al. (30)	196 (138/43/15)	Variable among studies.	Low dose (375 mg/m^2^ twice a month) vs. high dose (any other regimen).	Change in QMG.	Reduction of QMG: 4.16. No difference between Rituximab and Eculizumab.
Zhao et al. (27)	417 (242/155/20)	Variable among studies.	Most patients received 375 mg/m^2^ × 4 weeks or 1 g × 2 weeks. Variable regimens in the rest of patients.	Proportion of patients achieving MMs and change in QMG.	64% achieving MMS or better. Mean reduction of QMG 1.55.
Tandan et al. (26)	169 (99/57/7)	Variable among studies.	Most patients received 375 mg/m^2^ × 4 weeks or 500 mg × 2 weeks. Variable regimens in the rest of patients.	Proportion of patients achieving MMs.	70% between MuSK-positive patients but 30% between AChR.
Iorio et al. (19)	168 (91/70/7)	Variable among studies.	Most patients received 375 mg/m^2^ × 4 weeks or 500 mg × 2 weeks or 1 g × 2 weeks. Variable regimens in the rest of patients	Change in MGFA-PIS.	Response in 83.9%.
**Observational studies**					
Nelke et al. (3)	56	24	1 g × 2 weeks.	Compare the change in QMG after treatment with RTX and eculizumab	Greater benefit with eculizumab.
Li et al. (31)	19 (19/0/0)	51.3 (30.3–72.5)	Based on CD19 count at baseline and repopulation.	Change in QMG score.	Positive (median QMG decreased from 18 to 11).
Fatehi et al. (32)	34 (17/9/8)	12	1 g × 2 weeks and reinfusions every 6 months.	Change in average score on MGC, MGQoL-15, MGFA and MG-ADL. Change in prednisolone and pyridostigmine doses.	Improvement in MG-QoL and MGC. Reductions in the average daily dose of both drugs.
Zhou et al. (33)	12 (0/12/0)	6	600 mg single infusion.	Change in QMG, MGC, MMT, MG-ADL y MG QOL-15.	Decrease in all scales.
Martínez-Monte et al. (34)	20 (16/2/2)	31,7 (15,2)	NA	Clinical response (complete/partial/absence).	75% of response (60% complete).
Doughty et al. (35)	40 (28/9/3)	12	375 mg/m^2^ × 4 weeks or 1 g × 2 weeks.	Proportion of patients reaching a “Improved” or better in MGFA-PIS.	76.9% achieved primary endpoint.
Litchman et al. (36)	33 (17/16/0)	62.1 (31.8)	375 mg/m^2^ × 4 weeks.	Change in median MFGA and proportion of patients achieving MMs or better at 12 months and last visit.	MGFA change from II to 0 and MMs or better was attained in 64.7% (AChR+) and 75% (MuSK+).
Choi et al. (37)	17 (9/6/2)	24.5 (11.3)	375 mg/m^2^ × 2 weeks.	Achieving MMs or better in MGFA-PIS with prednisolone dose ⩽5 mg/day.	65% achieved primary endpoint.
Lu et al. (38)	12 (12/0/0)	18	600 mg every 6 months.	Change in QMG.	Decreased from 18.25 ± 4.03 to 8.42 ± 3.99.
Topakian et al. (39)	56 (39/14/3)	20 (10–53.5)	Most patients received 375 mg/m^2^ × 2 weeks or 500 mg × 2 weeks. Variable regimens in the rest of patients	MGFA-PIS.	MMs or better 67.9% at last follow-up.
Roda et al. (40)	27 (10/13/4)	NA	375 mg/m^2^ × 4 weeks or 1 g × 2 weeks.	(1) Efficacy of stopping conventional immunosuppressants. (2) Reduction in steroids daily dose. (3) Improvement in MGFA-PIS.	(1) Discontinuation in all the cohort. (2) Decreased from an average of 19.9 to 10.2 mg/day. (3) MMs or better in 55.5%.
Beecher et al. (25)	22 (10/9/3)	28.8 (19.0)	375 mg/m^2^ × 4 weeks + two reinfusions of 750 mg within 2 months.	Change in the MMT.	Mean reduction in MMT score from 10.3 to 3.3.
Landon-Cardinal et al. (41)	11 (11/0/0)	18	1 g × 2 weeks.	Improvement of at least 20-points on the MMS at 12 months.	Only one patient (9%) achieved primary endpoint.
Cortes-Vicente et al. (42)	25 (0/25/0)	60 (39.6)	375 mg/m^2^ × 4 weeks or 375 mg/m^2^ × 4 weeks + two reinfusions monthly or 1 g × 2 weeks.	Proportion of patients achieving MMs or better in MGFA-PIS.	Primary endpoint achieved in 100%.
Afanasiev et al. (21)	28 (21/3/4)	27.2 (16.6)	375 mg/m^2^ × 4 weeks or 1 g × 2 weeks.	Change in MGFA-PIS.	50% achieved Improved Status.
Hehir et al. (24)	24 (0/24/0)	45 (6∼116)	375 mg/m^2^ × 4 weeks.	MGSTI score.	58% achieved MGSTI level 2 or better.
Robeson et al. (43)	16 (16/0/0)	56.1 (20.1)	375 mg/m^2^ × 4 weeks.	MGFA-PIS.	63% achieved CSR; 19% Pharmacological Remission; 19% MMs.
Blum et al. (44)	14 (11/3/0)	14.4 (11.3)	500 mg × 2 weeks.	Change in MGFA-PIS.	78.5% achieved Improved or better.
Maddison et al. (45)	10 (7/3/0)	12 ∼ 48	375 mg/m^2^ × 4 weeks.	MGFA-PIS.	25% of CSR and 33% of MMs or Improved.
Díaz-Manera et al. (46)	17 (11/6/0)	31	375 mg/m^2^ × 4 weeks.	MGFA-PIS.	100% of MuSK+ achieved MMs. 90.9% of AChR+ achieved Improved.
Collongues et al. (23)	13 (8/3/2)	26 (13)	375 mg/m^2^ × 4 weeks or 1 g × 2 weeks.	ARR and MGFA scores.	Decrease ARR from 2.1 to 0.3 and lower MGFA scores in both refractory and non-refractory patients.
Nowak et al. (20)	14 (6/8/0)	12	375 mg/m^2^ × 4 weeks.	Change in corticosteroids dose.	Prednisone dose decreased a mean of 93.8% after cycle 3 of RTX.

SNMG, Seronegative myasthenia gravis; RTX, Rituximab; QMG, Quantitative myasthenia gravis; MMs, Minimal manifestations status; MGFA-PIS, Myasthenia Gravis Foundation of America—Postinterventional Status; MGC, Myasthenia gravis composite; MGQoL-15, Myasthenia gravis quality of life 15; MGFA, Myasthenia Gravis Foundation of America; MG-ADL, Myasthenia gravis activities of daily living; MMT, Manual muscle testing; MMS, Myasthenic muscle score; MGSTI, Myasthenia gravis status and treatment intensity; ARR, Annualized relapse rate; CSR, Complete stable remission.

However, all these studies have often reported contradictory results and differences among different subgroups of autoantibodies. Other questions such as the adequate dosage, monitoring strategies, re-infusion regimen or long-term security profile remain also unclear.

### 4.1. Rituximab treatment in patients with anti-AChR antibodies

#### 4.1.1. Evidence of use

During the decade of 2010, several observational studies were published reporting good responses of patients with AChR-positive MG patients and reductions in the corticosteroid doses after treatment with Rituximab (21–23). Although those studies pointed toward a treatment benefit, the evaluated outcomes and population baseline characteristics were different between studies and results exhibited high variability in the degree of improvement.

In a single-center study of patients with refractory MG treated with Rituximab published in 2017, the impact on patients’ quality of life and the difference in annual healthcare costs per patient compared to the year before rituximab initiation were analyzed, showing a favorable cost-effectiveness balance (47).

In the same year, a meta-analysis of the evidence on the use of Rituximab in MG was published, including a total of 169 patients. Among patients with positive anti-AChR antibodies, it was observed that 30% of patients achieved a status of minimal manifestations or better on the Myasthenia Gravis Foundation of America—Post Interventional Status score (MGFA-PIS) and a 46% reduction in the Quantitative Myasthenia Gravis score (QMG) (26).

Two new meta-analyses have been published in recent years, including patients with refractory MG and positive anti-AChR antibodies treated with rituximab. Both showed a proportion of patients reaching a state of minimal manifestations or better on the MGFA-PIS scale after Rituximab treatment of 51% (27) and 54% (48).

However, in 2018, the first randomized, double-blind, placebo-controlled clinical trial in MG patients with anti-AChR antibodies treated with Rituximab (BEAT-MG) was conducted and published in 2022. A non-inferiority design was used, with the primary efficacy objectives being the reduction in daily corticosteroid dose and the score on the MGC scale. This objective was achieved by 60% of the patients, a rate of benefit which is consistent with the results of previous studies. However, there was a high percentage of patients in the placebo group who also achieved this objective (56%), so the study did not show statistically significant differences between placebo and Rituximab in these patients. Moreover, no differences were found in quantitative scales such as QMG or MGC scores (49).

On the other hand, at the end of 2022, the results of the RINOMAX trial were published, a randomized, double-blind, placebo-controlled study in which 47 patients (45 of whom were positive for anti-AChR antibodies, and only two were MuSK-positive) were randomized to receive Rituximab vs. placebo. The primary objective was the proportion of patients with minimal manifestations, defined as QMG < 4 with a prednisolone dose of less than 10 mg/day. This was achieved by 71% of patients with rituximab compared to 29% of patients with placebo. A lower need for hospitalizations and rescue treatments with immunoglobulin or plasmapheresis in the rituximab group, as well as a lower corticosteroid dose at the end of the study in the rituximab group were also found. However, the study did not meet the secondary objectives of reducing QMG and MG-ADL scores, although patients who received rescue treatments during the study were excluded from the analysis, which had a greater impact on the placebo group, affecting the power to detect differences between arms.

Another limitation of this trial was an imbalance in important characteristics of the baseline populations in the two arms. Patients in placebo group were younger, had higher titers of antibodies and were taking lower doses of oral corticosteroids. More patients were classified as MGFA III in the placebo group whereas in Rituximab most of them were classified as II. Although predictive factors of response to treatment remain unknown, those are important characteristics which might had impacted on results.

In a recent meta-analysis published in 2023, including clinical trials of new therapies for myasthenia gravis, rituximab was the only one of the three analyzed strategies (anti-CD20, anti-FcRn, and anti-complement) that did not show statistically significant differences compared to placebo in reducing scores on the MG-ADL, MGC, QMG, and patients’ quality of life. However, as the authors themselves noted, none of these scales were the primary outcome of the trials and the number of recruited patients was significantly lower than for the rest of therapies, giving place to large CI (6).

This discordance among the results of different observational studies, clinical trials, and meta-analyses can be attributed to significant heterogeneity in the methods and conduct of the studies, with considerable variability in the analyzed variables as objectives, which complicates comparisons. This encourages the need to conduct new clinical trials with a uniform methodology, where primary objectives were common, and baseline characteristics of the study population were carefully recorded.

#### 4.1.2. Recommendation of use

In the latest international consensus guidelines for the management of MG updated in 2021, the efficacy of rituximab in patients with anti-AChR antibodies is still considered uncertain, although its use is considered an acceptable alternative in refractory cases (50).

### 4.2. Rituximab treatment in patients with anti-MuSK antibodies

#### 4.2.1. Evidence of use

Patients with anti-MuSK antibodies constitute approximately 5% of all MG cases (2). These patients have been associated with a bulbar phenotype of the disease and with increased severity and refractoriness to other immunosuppressive treatments and intravenous immunoglobulin. This has been highlighted in studies showing a higher proportion of patients in the MuSK positive group requiring second-line immunosuppressive therapies such as Rituximab (22).

To the best of our knowledge, in 2006 was reported the first case of MuSK-positive MG patient successfully treated with Rituximab (51). Isabel Illa et al. published a series of 6 patients treated with Rituximab, highlighting a differential response between patients with anti-AChR antibodies and MuSK-positive, with greater and more sustained responses in this last group (22). In the light of these findings, the same group conducted a retrospective study comparing the results of Rituximab treatment between different serologic groups in 17 MG patients, confirming the better and lasted longer response in the MuSK-positive group (46).

In 2014, the first meta-analysis on the use of Rituximab in patients with MG was published. However, in this article, although the authors point to better outcomes among MuSK-positive patients, differences were minimal (88.8% improvement in MGFA-PIS vs. 85.6% in anti-AChR) and did not reach statistical significance (19). Subsequent studies have supported this evidence, finding greater differences between the two groups, suggesting that MuSK-positive patients benefit more from rituximab therapy, showing a faster response, tolerate a greater reduction in oral immunosuppressants and corticosteroids, and take longer to relapse (25, 27, 36, 52).

Also, in a multicenter retrospective study of all patients treated with Rituximab in the healthcare system of Austria, MuSK positivity was identified as the only independent variable associated with response to Rituximab (39). This finding was subsequently confirmed in another similar meta-analysis (26).

Despite the lack of clinical trials specifically focusing on the use of rituximab in MuSK positive patients (only 2 patients were included in RINOMAX), evidence from observational studies supports the same line of findings. In a prospective multicenter, double-blind study comparing MuSK-positive MG patients treated with rituximab vs. other conventional immunosuppressants, better responses and lower corticosteroid doses were demonstrated in the intravenous treatment arm (24).

#### 4.2.2. Recommendation of use

Currently, international consensus guidelines for the management of MG consider Rituximab as an early-line treatment in MuSK-positive patients who have failed first-line immunotherapy (50).

### 4.3. Rituximab treatment in seronegative patients

#### 4.3.1. Evidence of use

The evidence for the use of Rituximab in seronegative Myasthenia gravis (MG) patients is scarce and limited to the inclusion of a small number of patients in observational studies.

In 2015 and subsequently in 2017, two meta-analyses were published that included 4 and 7 seronegative patients, respectively. While the first meta-analysis showed an 85.6% improvement in MGFA-PIS scale, the second meta-analysis reported improvement in only one patient (19, 26). In 2018, the results of a prospective study on the use of rituximab in refractory MG were published, which included three seronegative patients. The study did not demonstrate improvement in the primary outcome (reduction in Manual Muscle Testing score), although one of the three patients was able to discontinue corticosteroid therapy (25). In 2019, another retrospective study included 4 seronegative patients, of whom 3 showed Improvement status on MGFA-PIS after treatment with Rituximab. In all cases, corticosteroid-sparing therapy could be discontinued, and the prednisone dose was reduced in two patients (40). However, in the same year, another retrospective study based on the Austrian healthcare population did not show clinical remission in any of the three seronegative patients included in the study and treated with Rituximab (39). Finally, the study with the largest number of seronegative patients included is a meta-analysis published in 2021, which included 20 seronegative patients treated with Rituximab. 40% of these patients achieved a status of minimal manifestations or better, compared to 51% in anti-AChR patients and 79% in MuSK-positive patients. The reduction in the mean daily corticosteroid dose was also lower in this subgroup, although withdrawal of other immunosuppressive agents was observed in up to 91% of patients (27).

#### 4.3.2. Recommendation of use

The data to date is limited and contradictory in the literature. Although it is not possible to establish an evidence-based recommendation for the use of rituximab in these patients, there are reported cases of improvement in seronegative patients with refractory MG, which supports the use of rituximab in selected cases.

### 4.4. Rituximab treatment in juvenile myasthenia gravis

Juvenile Myasthenia Gravis (JMG) is defined as the onset of the disease in patients younger than 18 years old. It is a rare condition with an incidence of around 1.5 patients per million inhabitants per year and represents 3%–15% of all MG cases in Europe, while in Asia, it is estimated to reach up to 50% (53, 54). Pure ocular forms are more common in female patients with prepubertal onset, especially in the Asian population. The rate of generalization in JMG patients varies depending on the studies, ranging from approximately 25 to 35%, which is much lower than in adult patients. Generalized forms of JMG typically debut in the post-pubertal period (53–55). Another difference compared to adult patients is the prevalence of antibodies, with a high rate of seronegative patients (36%–50%) (54) and a higher rate of seroconversion during the course of the disease compared to adults (55).

Regarding the treatment of JMG, corticosteroids are the first-line immunosuppressive therapy. However, high rates of corticosteroid dependence have been reported among pediatric patients (54). In refractory cases or when symptoms reappear and corticosteroid dose reduction is not possible, second-line immunomodulatory therapies are indicated.

#### 4.4.1. Evidence of use

The experience with Rituximab in JMG is limited to case reports, generally showing favorable outcomes in patients with seropositivity for anti-AChR and anti-MuSK antibodies, as well as in seronegative patients (56–61).

Two subsequent larger studies documented the experience with five patients each (seven patients with anti-AChR antibodies and three MuSK-positive patients), all of whom showed improvement (54, 62). Despite the improvement, two children with anti-MuSK antibodies required high doses of corticosteroids to maintain their condition (54).

A recent multicenter retrospective study conducted in several French hospitals included 27 pediatric patients treated with Rituximab (63). The patients treated with Rituximab showed better outcomes compared to other conventional therapies, allowing for a reduction in corticosteroid doses and withdrawal of immunosuppressants. No adverse events were reported during the study.

#### 4.4.2. Recommendation of use

Currently, the evidence is limited, but the results indicate that Rituximab is a well-tolerated and effective option in the treatment of JMG.

## 5. The physiopathology behind the evidence

After antigen recognition by B-Cell Receptors (BCR), activated B cells give rise to short-lived plasmablasts and plasma cells, which are effectors of the initial humoral response. The sustained exposure to the antigen eventually leads to the development of long-lived plasma cells. On the other hand, antigen-activated B cells can undergo affinity maturation of the BCR to give rise to memory B cells, which, along with long-lived plasma cells, provide long-term immunity (64).

As we said before, Rituximab causes depletion of circulating B cells and a 70% reduction of this lineage in narrow bone, including plasmablasts, thus suppressing the initial humoral response. Rituximab also produces depletion of memory B cells, leading to repopulation of the periphery with naive B cells and generating an altered balance between different cell populations and their activity. Some evidence has emerged showing how administration of Rituximab also leads to an increased percentage of the T regulator lymphocytes (11, 65).

This abortive effect over the first humoral immunity has several implications:

On one side, this is probably the reason behind the differential response to Rituximab between MuSK and AChR-positive MG patients. Anti-MuSK antibodies are predominantly of the IgG4 subtype which are thought to be mainly produced by short-lived B cells and plasmablasts, a pool of cells that requires constant replenishment from B cell precursors and which are depleted after Rituximab administration, justifying the better and sustained response in these patients (52).

This evidence is supported by the quick reduction in titers of MuSK antibody after the treatment (26, 46). Elevations in anti-MuSK antibody titers have also been described in patients who experience relapses. In a study analyzing samples from patients who had relapsed following initial improvement after rituximab, the presence of specific memory B cells against MuSK and an increase in peripheral blood plasmablasts were demonstrated, as well as an increase in anti-MuSK antibody titers compared to patients who remained in complete remission or controls (66).

The other implication of depleting the effectors of the early humoral response is the possible impact of disease duration in the response to Rituximab. It is speculated that early administration of anti-CD20 therapies in autoimmune diseases may abort the process of forming autoantibody-secreting plasma cells, maintaining sustained therapeutic response (64, 65, 67).

This could explain the differences between the two clinical trials in MG, since in RINOMAX, the included patients had 12 months or less of disease duration, while in BEAT-MG, there was no limit on disease duration (mean disease duration was 5.5 years). This is consistent with previous studies in which disease duration is the only factor correlated with a faster response to rituximab (19, 67).

## 6. Regimen of administration

There is no clear consensus regarding the appropriate administration regimen of Rituximab for patients with MG. The most commonly used guidelines are those indicated for patients with B-cell lymphoma, which consist of either weekly doses of 375 mg/m^2^ for 4 consecutive weeks or two doses of 1 g, with a 2-week interval between them (27).

In recent years, several authors have started to suggest the use of lower doses of Rituximab in patients with autoimmune diseases, as the lymphocyte burden is lower compared to patients with hematological neoplasms. These lower dose regimens have already proved efficacy in other autoimmune diseases (68). In MG, a meta-analysis by Li and colleagues reported that up to 34% of patients receive these reduced-dose regimens, which can vary greatly: two doses of 500 mg separated by 2 weeks, a single induction dose of 600 mg, two weekly doses of 375 mg/m^2^, etc. (69). However, results are contradictory. A meta-analysis comparing MG patients with anti-AChR antibodies who had received the standard dose vs. a reduced dose found no differences in clinical response, reduction in corticosteroid dosage, or withdrawal of conventional immunosuppressants (69). These findings are in line with previous studies, which also did not observe differences in the need for reinfusion (19, 26, 39). In contrary, another meta-analysis including 196 refractory MG patients showed a higher rate of Minimal Manifestations MGFA-PIS between the high-dose group of Rituximab (84% vs. 39%) (30). In another retrospective multicenter study of patients with anti-MuSK MG, a higher risk of relapse and shorter time to relapse were observed in patients who had received two weekly doses of 1 gram compared to patients who had received 6 doses of 375 mg/m2, with no differences in safety between both regimens (42).

Since the evidence reported to date are controversial, the question about the adequate dosage of Rituximab for MG patients remains still unclear.

## 7. Monitorization after treatment

Regarding maintenance therapy, there is no established protocol for the need for reinfusion. Several monitoring strategies have been proposed based on analytic markers. Since anti-AChR and anti-MuSK antibodies have a pathogenic role in the disease (22), monitoring titers of antibodies has been postulated as a possible option.

As for the AChR antibodies, neither of the two clinical trials conducted so far, proved a significant reduction in antibody titers after Rituximab treatment (49). Other observational studies did report a reduction in titers, although none of them demonstrated a correlation with clinical improvement, so monitoring these antibodies is not indicated as a predictor of the patient’s clinical course (26, 48).

In contrast to anti-AChR antibodies, anti-MuSK antibody titers do closely correlate with disease severity (47), with reductions of up to 90% in titers being described in patients who respond to rituximab administration (26, 46). Elevations in anti-MuSK antibody titers have also been described in patients who experience relapses compared to those who remained in complete remission (66).

As Rituximab treatment induces B cell depletion, B cell counts in peripheral blood has also been used to monitor in some studies and seems to have a better correlation with the clinical response than the antibodies titer (69). In several autoimmune diseases, including MG, monitoring CD20 cells have proved its value as a predictor of clinical relapses (70, 71). However, Choi and colleagues reported B-cell recovery appeared to be in parallel with clinical relapse on the group level, although it was not a good predictor at the individual-level, with B-cell repopulation observed only at 57% of clinical relapses (37).

In recent years, the levels of CD27+ cells, corresponding to memory B cells, have been described as possible monitoring marker, with a stronger correlation than antibody titers or total B lymphocytes. In a study by Lebrun et al., no patients with low levels of CD27+ cells experienced relapses, while an increase in the levels of these cells correlated with the appearance of symptoms in all cases (72). Using this marker to guide re-treatment decreased the number of annual cycles without a higher number of relapses. However, other authors have noted that, despite of a good sensitivity as a risk of relapse marker, only a 21% of patients with levels of CD27+ cells above threshold manifested a clinical worsening, which could lead to an overtreatment (71). The applicability of this marker in clinical practice is not yet well defined.

In literature, in most studies and reported cases, the decision to administer a new cycle is based on the reappearance of symptoms or the count of CD20+ cells in peripheral blood, while a minority of cases administered Rituximab on a periodic basis (39, 42, 69).

## 8. Safety profile

As previously mentioned, experience with Rituximab dates back to the last two decades, so there is abundant evidence regarding the drug’s safety.

In 2015, safety results of over 3,000 patients with rheumatoid arthritis treated with Rituximab were published, following an 11-year follow-up. The results showed that Rituximab does not pose a higher risk of serious adverse effects, including severe infections, cardiovascular events, or neoplasms (73).

In MG, the rate of adverse events reaches 15%–20% depending on the studies. In most cases, these are mild events that occur within the first 6 months of treatment and are related to infusion reactions, which can present as flushing, flu-like symptoms, fever, etc. (26, 27).

Another concern regarding the use of Rituximab is infections. In MG patients, a risk of serious infections of 0.05/100 patient-years has been reported, including respiratory and gastrointestinal infections, erysipelas, or herpes zoster reactivation. No increased risk of infections has been demonstrated with Rituximab compared to placebo (4).

Rituximab has been associated with the induction of hypogammaglobulinemia, which is associated with a higher risk of serious infections (73, 74). In a retrospective multicenter study of Rituximab-treated MG patients with anti-AChR and anti-MuSK antibodies, it was observed that 37% of patients developed hypogammaglobulinemia, of which 70% was mild. However, no association was demonstrated between hypogammaglobulinemia and the development of serious infections (74).

An infection that deserves special attention due to its poor prognosis is progressive multifocal leukoencephalopathy (PML), an opportunistic infection of the central nervous system caused by the John Cunningham (JC) virus. PML infection has been associated with the use of Rituximab with a frequency of 1 in 20,000 treated patients (16). To date, three confirmed cases of PML have been reported in MG patients associated with the use of Rituximab, although all of them had previously received other conventional immunosuppressive therapies (21, 74, 75).

On the other hand, resistance to Rituximab mediated by inhibitory human anti-chimeric antibodies has been described in 1% of patients with hematological malignancies. As far as we know, only one case of resistance to Rituximab have been reported in MG patients to date. A 28 years old female patient who was tested for these antibodies due to absence of response after the third infusion of Rituximab (26, 74).

In summary, Rituximab is considered a safe alternative in MG patients, with a complication rate similar to other immunosuppressants (27).

## 9. Discussion

Since the first use of Rituximab in a patient with MG in 1999, a large number of studies have shown benefits of this therapy over the past 20 years. However, results between studies have often been inconsistent, and it has been suggested that the treatment’s efficacy depends on the patient’s serotype.

Currently, the benefit of Rituximab therapy in MuSK-positive patients seems clear. Although no clinical trials have been conducted in this patient subgroup, accumulated evidence from observational studies and meta-analyses over the past few decades has been consistent and has shown a positive effect not only in the clinical improvement of these patients but also as a steroid-sparing and other immunosuppressive medications, making Rituximab a therapeutic option even ahead of conventional immunosuppressants (27, 36, 46, 50).

The situation is different, however, for AChR-positive seropositive patients. The high variability in the results of observational studies is evident in systematic reviews, which have shown benefits ranging from 30% to 54% to negative results (6, 26, 27). In addition, two recently published randomized clinical trials have been conducted in these patients. The first trial, BEAT-MG, concluded with a treatment futility outcome. The second trial, the RINOMAX trial, has generated controversies because, despite achieving the primary endpoint, the baseline populations of both arms were significantly different in terms of age, corticosteroid dose, and disease severity. Furthermore, this trial was negative in the secondary outcomes (49).

Therefore, with the available evidence to date, it is not possible to ascertain a clinical benefit of Rituximab in AChR-positive patients, and in our opinion, the use of Rituximab in these patients should be restricted to refractory cases and after individualized therapeutic decision-making.

Another issue yet to be clarified is the appropriate administration regimen in these patients. Although most studies have been conducted following classical treatment guidelines (375 mg/m^2^ × 4 weekly doses or 1 g × 2 fortnightly doses), in recent years, an increasing number of authors have suggested that lower doses of Rituximab achieve the same clinical effect with a better safety profile and cost-effectiveness (26, 39, 69).

This uncertainty in the administration regimen also applies to the re-infusion schedule, and there is an increasing need for a marker that guides monitoring and retreatment before clinical relapse occurs. Unlike anti-AChR antibody titers, it is clear that anti-MuSK antibody titers have a good correlation with patient symptomatology and can predict relapses in the majority of cases (26, 46). Other markers that have been proposed as promising in recent years are the count of CD27-positive memory B cells (72). However, in neither case are there guidelines that apply this correlation in clinical practice.

Finally, Rituximab appears to be a safe long-term therapy for patients with MG. In general, most adverse effects are related to infusion reactions, and there has been no demonstrated increase in the risk of serious infections or neoplasms in these patients (27).

Despite the extensive experience accumulated over 20 years of using this treatment, many questions remain unresolved. In our opinion, new clinical trials are needed to clarify the question of efficacy in anti-AChR-positive patients, conducted with a stratification process that ensures similarity between both arms of the trial, as well as new guidelines that standardize the use of Rituximab regimen among different studies and centers and allow the implementation of new markers in the monitoring of these patients.

## Author contributions

AV-C: Writing – original draft, Writing – review & editing, Conceptualization, Methodology. EC-V: Writing – original draft, Writing – review & editing, Conceptualization, Funding acquisition, Methodology, Supervision.
